# High Glucose Induces Autophagy through PPAR*γ*-Dependent Pathway in Human Nucleus Pulposus Cells

**DOI:** 10.1155/2018/8512745

**Published:** 2018-03-01

**Authors:** Chang Jiang, Shuhao Liu, Yuanwu Cao, Hongping Shan

**Affiliations:** ^1^Department of Orthopaedics, Zhongshan Hospital, Fudan University, Shanghai, China; ^2^Department of Nephrology, Pudong Medical Center, Fudan University, Shanghai, China

## Abstract

Diabetes mellitus is a multiorgan disorder affecting many types of connective tissues, including bone and cartilage. High glucose could accelerate the autophagy in nucleus pulposus (NP) cells. In our present study, we investigated whether peroxisome proliferator-activated receptor *γ* (PPAR-*γ*) pathway is involved into autophagy regulation in NP cells under high glucose condition. After NP cells were treated with different high glucose concentrations for 72 hours, the rate of autophagy increased. Moreover, the levels of PPAR*γ*, Beclin-1, and LC3II were significantly increased and p62 was significantly decreased compared to control group. Then, NP cells were treated with high glucose plus PPAR*γ* agonist or PPAR*γ* antagonist, respectively. The rate of autophagy and the levels of Beclin-1 and LC3II increased, but p62 decreased when PPAR*γ* agonist was used. On the contrary, the rate of autophagy and the levels of Beclin-1 and LC3II decreased, while p62 increased when PPAR*γ* antagonist was added. These results suggested that autophagy induced by high glucose in NP cells was through PPAR*γ*-dependent pathway.

## 1. Introduction

Low back pain is highly prevalent and accounts for low life quality, and it is widely accepted that back pain has relationship with lumbar disc degeneration (LDD). Although the strength of the association is still controversial [1, 2], deterioration of intervertebral disc (IVD) could lead ultimately to the development of symptomatic degenerative disorders such as disc herniation, spinal stenosis, and degenerative spondylolisthesis.

Many evidences showed that diabetes mellitus (DM) is important in the aetiology of LDD. It was reported that there is a higher incidence of degenerative disc diseases in patients with DM than in the non-DM population [3] and patients with DM had poorer surgical outcomes versus non-DM patients [4].

The decrease in the number of viable nucleus pulposus (NP) cells in diabetes was thought to be one of the initial triggers of disc degeneration. In vitro studies, high glucose has been proved to accelerate autophagy [5], apoptosis [6], and senescence [7] in adult rat NP cells in a dose- and time-dependent manner. Both apoptosis and senescence of IVD cells can lead to the decrease of viable cells. The role of autophagy is relatively complex. Initially, autophagy can promote cell survival and avert apoptosis during stress responses by turning over the intracellular organelles and molecules through the lysosomal pathway. However, when autophagy is prolonged, proteins and organelles essential for basic homeostasis and cell survival are degraded, which can lead to cell death. But the mechanism of autophagy in NP cell under high glucose is still unclear.

Peroxisome proliferator-activated receptors (PPAR) are a kind of ligand-activated transcription factors that regulate vital genes in cell differentiation and various metabolic processes. The family of PPARs in mammals comprises three isoforms: PPAR*α*, PPAR*β*/*δ*, and PPAR*γ* [8]. Activation of PPAR*α* induces lipid metabolism and regulates energy homeostasis, whereas the activation of PPAR*β*/*δ* enhances fatty acid metabolism. On the other hand, activation of PPAR*γ* leads to an improvement of insulin resistance, followed by glucose metabolism.

PPAR*γ* is expressed in white and brown adipose tissue, the large intestine, and spleen. A plethora of evidence implicates that PPAR*γ* plays a central role in numerous diseases, including obesity, diabetes, and atherosclerosis [9–11]. But up to now, PPAR*γ* has never been reported in the NP cells and in intervertebral disc degeneration in previous literature.

Giving the role of PPAR*γ* in glucose metabolism and the role of high glucose in intervertebral disc degeneration, we supposed that the high glucose condition induces autophagy via PPAR*γ*-dependent pathway in our present study.

## 2. Methods

### 2.1. Cell Preparation

An intervertebral disc (L45) was harvested from a forty-four-year-old female patient who accepted transforaminal lumbar interbody fusion operation because of lumbar disc herniation. The disc degeneration was grade III according to Pfirrmann classification. The disc was dissected carefully under a microscope to obtain only the NP tissues under aseptic condition. Then, the harvested NP tissues were cut as small as possible(<1 mm3), digested with 0.25% type II collagenase for 4 h, and filtered with a sieve of 70 microns. After being washed with phosphate-buffered saline (PBS) twice, the NP cells were cultured in a culture flask with DMEM low-glucose medium supplemented with 10% fetal bovine serum (FBS) and 1% penicillin-streptomycin (Gibco BRL) at 37°C in a humidified atmosphere (95% air, 5% CO2). Medium was changed two or three days later. When the cells reached 80%–90% confluence, they were split once (passage 1) and grown to 80%–90% confluence again. The cells after 3 passages were used for subsequent experiments.

### 2.2. Cell Culture

The cells after 3 passages were cultured in DMEM containing 5.6 mM (normal control), 0.1 or 0.2 M glucose, supplemented with 10% fetal bovine serum, and 1% penicillin-streptomycin (Gibco BRL) and maintained at 5% CO2 on poly-L-lysine coated plates. For pharmacologic intervention of glucose-induced cell change, cells at 0.2 M glucose medium were switched to 0.2 M glucose medium with 25 uM/L T0070907 or rosiglitazone (Selleck Chemicals, Shanghai, China), respectively.

### 2.3. Western Blot

NP cells were harvested for protein quantification by using RIPA (Beyotime, Shanghai, China) with 1% phenylmethanesulfonyl fluoride (PMSF). Protein concentrations were assayed by a BCA kit (Piece, Biotechnology, MA, USA). Protein sample was electrophoresed on 12% polyacrylamide gel in the presence of SDS-PAGE and transferred onto a polyvinylidene difluoride membrane (millipor). Blots were blocked for 1 h at room temperature with 5% nonfat dry milk in TBST buffer. Afterward, the membranes were incubated with the primary antibodies against LC3II/I (CST, 1 : 1000); Beclin1 (CST, 1 : 1000); PPAR*γ* (Proteinteck, 1 : 500); p62 (CST, 1 : 10000); and GAPDH (Abcam, 1 : 10000) overnight at 4°C and HRP-conjugated secondary antibodies (CST, 1 : 2000) at room temperature. The bands were visualized by using ECL substrate (ThermoFisher) and analyzed by ImageJ software. The protein expression was normalized to control GAPDH levels.

### 2.4. Real-Time PCR

After being washed with PBS, total RNA was extracted from NP cells by using TriZol reagents (Invitrogen, California, USA) by following the manufacturer's protocol and its absorbance at 260/280 nm was detected to evaluate the RNA quality. The absorbance of 1.8–2.0 was considered to be good. cDNA was synthesized using SuperScript™ RT reagent kit with gDNA Eraser (Takara, Shiga, Japan) from 1 ug RNA. For PCR amplification, 10 ul of reaction volume included 5 ul of 2Power SYBR Green PCR Master Mix (ABI, Waltham, Massachusetts, USA), 0.25 ul each primer, 1 ug cDNA, and 3.5 ul sterile distilled water. The cycle threshold (Ct) values were collected and normalized to the housekeeping gene GAPDH. The 2^−ΔΔCT^ method was used to calculate the relative mRNA levels of each target gene. The primers for PPAR*γ* were 5′-TACTGTCGGTTTCAGAAATGCC-3′ (forward) and 5′-GTCAGCGGACTCTGGATTCAG-3′ (backward).

### 2.5. Fluorescence Microscopy

NP cells were cultured and grown to 80% confluence in 24-well plate. Appropriate amount of virus fluid containing packaged pL-CMV-TO-GFP-HLC3B (constructed by ourselves) was added to the medium. NP cells were incubated for 6 h at 37°C. The medium containing virus was replaced with DMEM supplemented with 10% fetal bovine serum (FBS) and 1% penicillin-streptomycin (Gibco BRL) and the NP cells were incubated for another 30 h at 37°C. Then, these transfected NP cells were treated as described in the section of cell culture. At last, autophagy in NP cells was observed and evaluated by fluorescence microscope. Autophagy rates were calculated by the number of autophagy cells over total ones under 10x lens.

### 2.6. Statistics

All experiments were repeated at least three times. The data were presented as means ± SD (standard deviation). SPSS 21.0 was used for all statistical analyses of the data. Comparison of data was performed using the one-way ANOVA test. Differences were considered statistically significant at *p* < 0.05.

## 3. Results

### 3.1. High Glucose-Induced Autophagy in NP Cells

**Figure 1:**
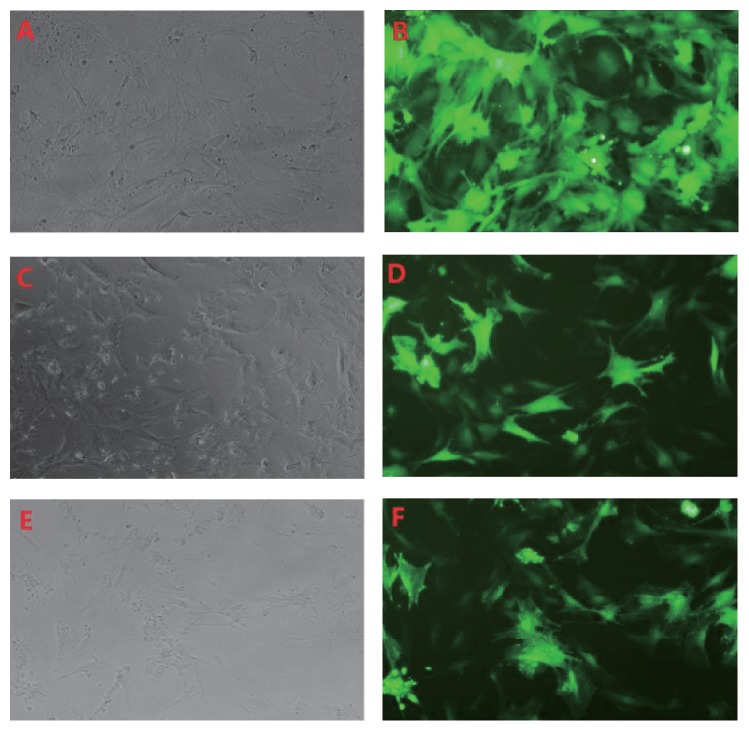
GFP-LC3B tracer technique showed that autophagy was induced in NP cells treated with high glucose condition ((A, C, E) light microscope; (B, D, F) fluorescence microscope. (A) and (B) represent glucose concentration as 5.6 mM; (C) and (D) as 0.1 M; (E) and (F) as 0.2 M, resp.).

**Figure 2:**
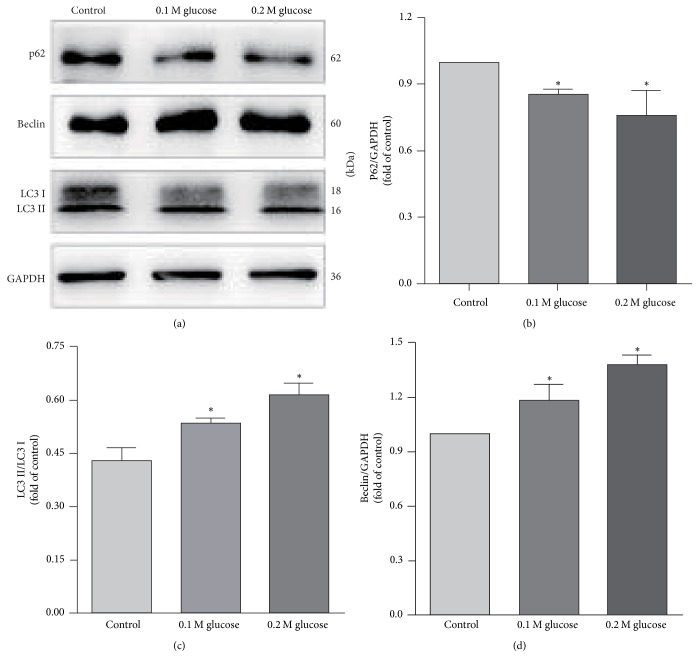
Western blot showed an increased expression of Beclin-1 and LC3-II and a decreased expression of p62 in the NP cells treated with high glucose. *∗* represents a significant difference from the control group (*p* < .05).

The NP cells were divided into three groups as control group, 0.1 M high glucose group, and 0.2 M high glucose group according to glucose concentration(5.6 mM, 0.1 M, and 0.2 M). To evaluate the role of high glucose on autophagy in NP cells, we next not only display the autophagy cells using GFP-LC3B tracer technique but also examine and compare the LC3, Beclin-1, and p62 expression in the NP cells in all three groups by western blot. Beclin-1 and microtubule-associated protein-1 light chain 3 (LC3) are required for autophagosome formation and maturation, one of the important steps for autophagy. p62 protein called the autophagy-specific substrate can be degraded together with LC3-II. The impaired autophagy is accompanied by accumulation of p62. GFP-LC3B would form spots when autophagy occurs. And the more spots, the more autophagy. By using GFP-LC3B tracer technique, the rate of autophagy in NP cells was 6.67 ± 0.86%, 8.15 ± 0.85%, and 10.57 ± 1.21% in control group, 0.1 M glucose group, and 0.2 M glucose, respectively (Figure 1). The rate of autophagy increased with the increase of glucose concentration. And the western blot analysis showed an increased expression of Beclin-1 and LC3-II and a decreased expression of p62 in the NP cells treated with high glucose concentrations when compared with the control group (Figure 2). The ratio of LC3-II/LC3-I expression increased under high glucose conditions, too. Moreover, it was also shown that the expression of Beclin-1, LC3-II, and p62 was changed almost in a glucose concentration-dependent manner.

### 3.2. PPAR*γ* Was Expressed in NP Cells and Upregulated by High Glucose

**Figure 3:**
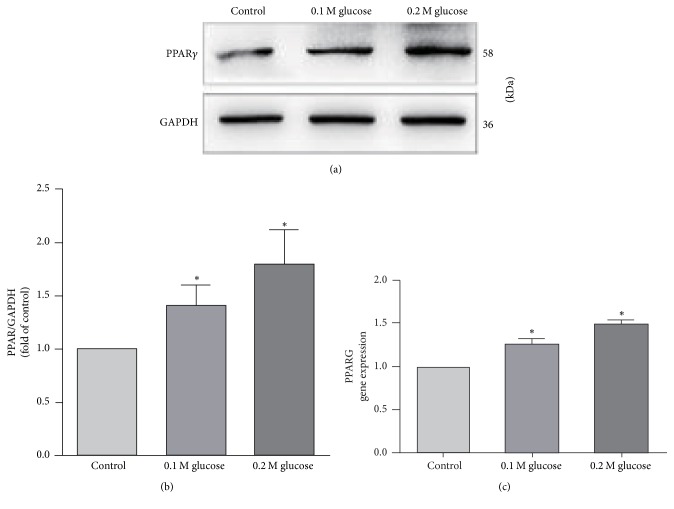
Real-Time PCR and western blot showed that PPAR*γ* expressed in the NP cells and the expression of PPAR*γ* increased when NP cells were treated with high glucose. *∗* represents a significant difference from the control group (*p* < .05).

Although PPAR*γ* has been confirmed to be expressed in many tissues, it has never been studied in NP cells. In order to evaluate whether NP cells also express the PPAR*γ* and the role of high glucose on PPAR*γ* expression, Real-Time PCR and western blot were used. By PCR and western blot examination, the presence of PPAR*γ* was detected at both RNA and protein levels in NP cells. When compared with the control group, it showed that the expression of PPAR*γ* increased in the NP cells treated with high glucose and the increase of PPAR*γ* content was almost paralleled with the elevation of glucose concentration (Figure 3).

### 3.3. PPAR*γ* Activation Induced Autophagy in NP Cells

**Figure 4:**
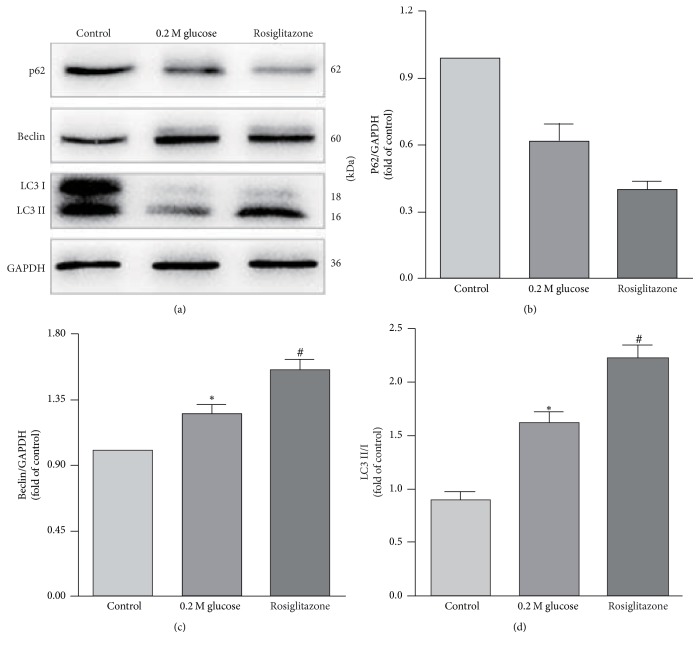
Western blot analysis showed an increased expression of Beclin-1 and LC3-II and a decreased expression of p62 in the NP cells treated with PPAR*γ* agonist. # represents a significant difference from 0.2 M high glucose condition (*p* < .05).

**Figure 5:**
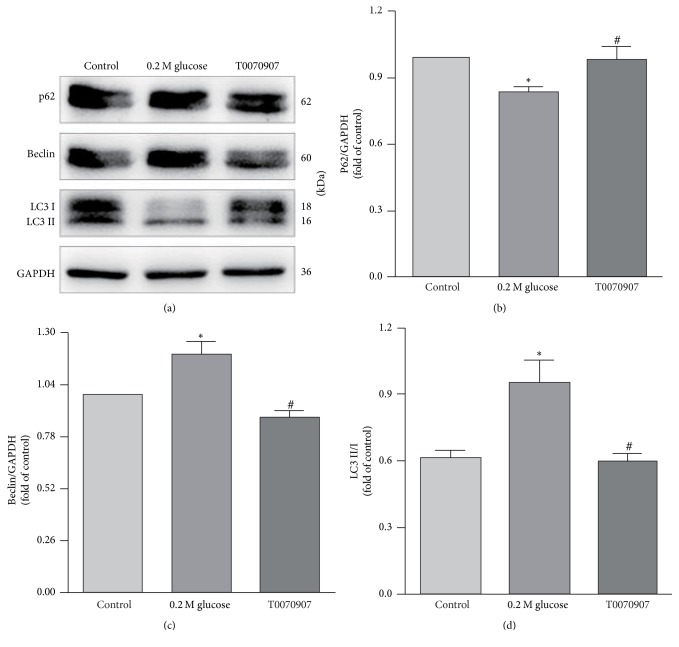
Western blot showed that the expression of Beclin-1 and LC3-II decreased and p62 increased in the NP cells when treated with PPAR*γ* antagonist. # represents a significant difference from 0.2 M high glucose condition (*p* < .05).

Considering that high glucose not only induced autophagy but also upregulated the expression of PPAR*γ* in NP cells, it was supposed by us that the PPAR*γ* should have a relationship with autophagy in NP cells under high glucose. To better understand this relationship, PPAR*γ* agonist and antagonist were added to the 0.2 M high glucose medium. Then the LC3, Beclin-1, and p62 expressions in the NP cells were examined and compared by western blot again. The western blot analysis showed an increased expression of Beclin-1 and LC3-II and a decrease expression of p62 in the NP cells treated with PPAR*γ* agonist when compared with the 0.2 M high glucose condition (Figure 4). On the contrary, the expression of Beclin-1 and LC3-II decreased and the expression of p62 increased in the NP cells when treated with PPAR*γ* antagonist (Figure 5).

**Figure 6:**
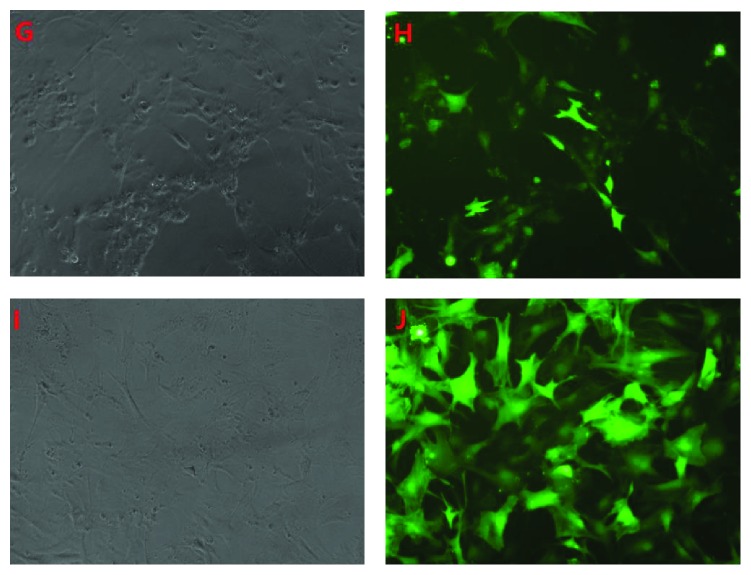
It showed that the autophagy in NP cells increased when the agonist was added to the high glucose medium, while it decreased when the antagonist was added ((G, I) light microscope; (H, J) fluorescence microscope. (G) and (H) represent as 0.2 M high glucose + agonist; (I) and (J) as 0.2 M high glucose + antagonist, resp.).

**Figure 7:**
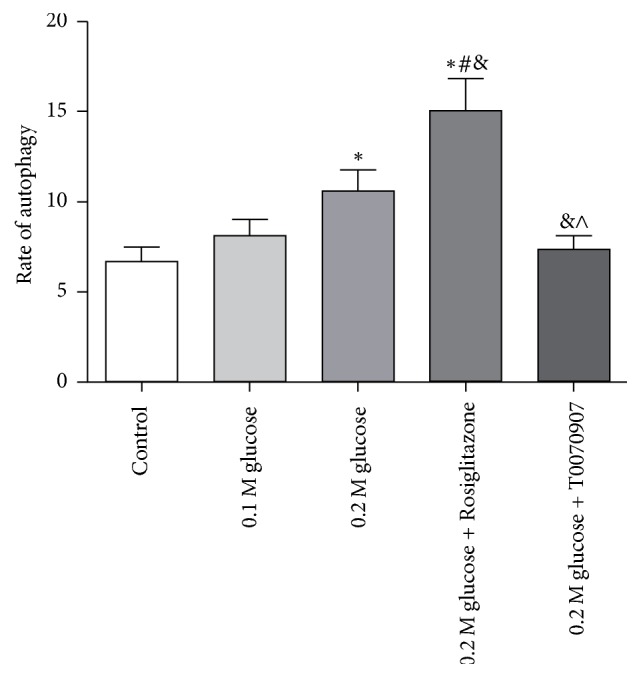
The rate of autophagy under different medium conditions was shown and the PPAR*γ* activation could induce autophagy significantly. *∗* represents a significant difference from the control group; # represents a significant difference from the 0.1 M glucose condition; & represents a significant difference from 0.2 M glucose condition; and ∧ represents a significant difference from 0.2 M high glucose condition plus agonist (*p* < .05).

Similarly, the autophagy in NP cells was also traced by using GFP-LC3B technique, when the agonist or antagonist was added (Figure 6). It showed that the autophagy in NP cells increased and the rate of autophagy was 15.08 ± 1.77% when the agonist was added. While the rate of autophagy decreased to 7.38 ± 0.75% when the antagonist was added, and it was almost the same as that in the control group (Figure 7).

## 4. Discussion

Glucose is an important fuel of nearly all organisms. It was used to be thought to lead to the elucidation of the electron transport chain and oxidative phosphorylation and complete the aerobic glucose metabolism for ATP generation through a common set of metabolic pathways. But now, it is known that glucose can be metabolized in multiple pathways providing not only an energy supply, but also participant in many important metabolites, such as cell growth and function. In recent years, high glucose has been proved to regulate autophagy in many cells, such as podocytes, renal tubular epithelial cells, and NP cells [5, 12, 13].

To be consistent with those previous results, our findings emphasize that high glucose could significantly enhance autophagy in NP cells. We have found both mRNA and protein levels of Beclin-1 and LC3B increased after high glucose treatment. Beclin-1 and LC3B are required for autophagosome formation and maturation, which is one of the most important steps of autophagy. We have also found that p62 protein decreased under high glucose. p62 is an autophagy-specific substrate, which can be degraded together with LC3-II. The accumulation of p62 indicated impaired autophagy. These findings indicated that the autophagy in NP cells activated by high glucose treatment and the autophagic flux was efficient. GFP-LC3B tracer technique also provided similar outcomes. When autophagy occurs, GFP-LC3B forms spots in cytoplasm. The more spots represents the higher level of autophagy. Our results showed that NP cells under 0.1 M and 0.2 M glucose resulted in more green spots than control, which revealed that glucose can certainly activate the autophagy in NP cells. However, the pathway of autophagy in NP cells under the high glucose condition is unclear.

PPAR*γ* is a nuclear hormone receptor that comprises an agonist-dependent activation domain, DNA binding domain, and agonist-independent activation domain. It is one of pathways in glucose metabolism involving the control of energy homeostasis, inflammation, proliferation, and differentiation [14]. It was reported that PPAR*γ* was expressed not only in adipose tissue [15, 16], but also in breast, colon, lung, ovary, prostate, and thyroid [17]. However, whether PPAR*γ* is expressed in human NP and how it can affect NP cells are unclear.

Here, it was the first time to identify that human NP cells also presented expression of PPAR*γ* by Real-Time PCR and western blot. Moreover, the expression of PPAR*γ* was found to be upregulated under high glucose treatment and the increase of PPAR*γ* was almost in direct proportion of the glucose concentration. Interestingly, the change pattern of PPAR*γ* in NP cells in our study varied with Schwann cells reported by Kim et al. [18]. The result in his study showed that chronic high glucose conditions retained normal PPAR levels in Schwann cells. The differences between Kim and us might be attributed to the differences in cell types, glucose concentration, and time of treatment.

There is no final conclusion about whether PPAR*γ* could activate or inactivate autophagy [19]. In our present study, PPAR*γ* was also found to have a relationship with autophagy in NP cells under high glucose condition and the increase of PPAR*γ* content was associated with the degree of autophagy. But whether the increase of PPAR*γ* is the upstream or the concomitant phenomenon of autophagy by high glucose needs be further studied. To better understand the relationship between PPAR*γ* and autophagy under high glucose condition in NP cells, the PPAR*γ* agonist and antagonist were added to DMEM with 0.2 M glucose. Our results revealed that PPAR*γ* agonist increased the autophagy in NP cells and the antagonist (T0070907) was opposite. According to these findings, we determined that PPAR*γ* was the upstream of autophagy and PPAR*γ* activation could further induce autophagy in NP cells under high glucose condition.

In summary, high glucose treatment could upregulate PPAR*γ*, which further activated autophagy in NP cells. Further observations are needed to uncover the specific signaling molecules on the PPAR*γ*-related autophagy induced by high glucose in NP.
